# 

**Published:** 1901-02

**Authors:** 


### BOOKS RECEIVED.

A Text-Book of Pharmacology and Therapeutics, or the Action of Drugs in Health and Disease. For the use of Students and Practitioners of Medicine. By Arthur R. Cushny, M. A., M. D., Aberd. Professor of Materia Medica and Therapeutics in the University of Michigan Medical Department, Ann Arbor. New second edition In one handsome octavo volume of 732 pages, with 47 engravings. Philadelphia and New York: Lea Brothers & Co. 1901. [Cloth, $3.75 net.]

Physical Diagnosis in Obstetrics. A guide in Antepartum, Partum and Post-partum Examinations for the Use of Physicians and Undergraduates. By Edward A. Ayers, M. D., Professor of Obstetrics in the New York Polyclinic; Attending Physician to the Mothers and Babies Hospital. Octavo, pp. 283. Illustrated. New York: E. B. Treat & Co. 1901. [Price, $2.00.]

Introduction to the Study of Medicine. By G. H. Roger, Professor Extra-ordinary in the Faculty of Medicine of Paris; Member of the Biological society; Physician to the Hospital of Porte D'Aubervilliers. Authorised translation by M. S. Gabriel, M. D., with editions by the author. Octavo, pp. 551. New York: D. Appleton & Co. 1901.

Transactions of the Medical Association of the State of Alabama. Annual meeting held at Montgomery, Ala., April 17-20, 1900. G. P. Waller, M. D., Secretary. Montgomery, Ala. Brown Printing Co. 1900.

Proceedings of the American Medico-Psychological Association. Fifty-sixth annual meeting held at Richmond, Va, May 23-25, 1900. C. B. Burr, M. D., Secretary. Published by the society. 1900.

Diseases of the Heart; Their Diagnosis and Treatment. By Albert Abrams, A. M., M. D., San Francisco, Consulting Physician for Diseases of the Chest, Mt. Zion Hospital and the French Hospital. Illustrated. Pages, 172. Chicago: G. P. Engelhard & Co. 1900. [Price, $1.00.]

Urinary Diagnosis and Treatment. By J. W. Wainwright, M. D., Member of the American Medical Association, New York State Medical Association, New York County Medical Association, etc. Illustrated with numerous engravings and colored plates, 140 pages. Chicago: G. P. Engelhard & Co. 1900. [Price, $1.00.]

Rudiments of Modern Medical Electricity. Arranged in the Form of Questions and Answers. Prepared Especially for Students of Medicine. By S. H. Monell, M D., New York, Professor of Static Electricity in the International Correspondence Schools; Founder and Chief Instructor of the New York School of Special Electro-Therapeutics. 12mo. pp. 165. New York: Edward R. Pelton, 19 E. 16th St. 1900. [Price, $1.00.]

Students' Edition, A Practical Treatise of Materia Medica and Therapeutics, with Special Reference to the Clinical Application of Drugs. By John V. Shoemaker, M. D., LL.D., Professor of Materia Medica, Pharmacology, Therapeutics, and Clinical Medicine and Clinical Professor of Diseases of the Skin in the Medico-Chirurgical College of Philadelphia; Physician to the Medico-Chirurgical Hospital. Fifth edition. Thoroughly revised Pages vii-770. Philadelphia: F. A. Davis Co. 1901. [Extra cloth, $4.00 net; Sheep, $4.75 net.]

A Clinical Treatise on Fractures. By William Barton Hopkins, M. D., Surgeon to the Pennsylvania Hospital and to the Orthopedic Hospital and Infirmary for Nervous Diseases. Octavo, pp. 268. Illustrated. Philadelphia: J. P. Lippincott Co. 1900.

An American Text-Book of Physiology. Edited by William H. Howell, Ph. D., M. D., Professor of Physiology in the Johns Hopkins University. Second edition, revised. Vol. II. Philadelphia and London: W. B. Saunders & Co. 1901. [Price, cloth, $6.00 net; Sheep or half Morocco, $7.00 net.]

Experimental Research into the Surgery of the Respiratory System. An Essay awarded the Nicholas Senn prize by the American Medical Association for 1898. By George W. Crile, A. M., M. D., Ph. D., Professor of Clinical Surgery Western Reserve University; Surgeon to St. Alexis Hospital; Associate Surgeon to Lakeside Hospital, Cleveland. Octavo, pp. 114. Second edition. Philadelphia: J. P. Lippincott Co. 1900.

